# *Plasmodium malariae* Infection Associated with a High Burden of Anemia: A Hospital-Based Surveillance Study

**DOI:** 10.1371/journal.pntd.0004195

**Published:** 2015-12-31

**Authors:** Siobhan Langford, Nicholas M. Douglas, Daniel A. Lampah, Julie A. Simpson, Enny Kenangalem, Paulus Sugiarto, Nicholas M. Anstey, Jeanne Rini Poespoprodjo, Ric N. Price

**Affiliations:** 1 Global and Tropical Health Division, Menzies School of Health Research and Charles Darwin University, Casuarina, Darwin, Northern Territory, Australia; 2 Division of Medicine, Christchurch Hospital, Christchurch, New Zealand; 3 Timika Malaria Research Program, Papuan Health and Community Development Foundation, Timika, Papua, Indonesia; 4 Centre for Epidemiology and Biostatistics, Melbourne School of Population and Global Health, University of Melbourne, Melbourne, Australia; 5 Mimika District Health Authority, Timika, Papua, Indonesia; 6 Rumah Sakit Mitra Masyarakat, Timika, Papua, Indonesia; 7 Division of Medicine, Royal Darwin Hospital, Darwin, Australia; 8 Department of Child Health, Faculty of Medicine, University Gadjah Mada, Yogyakarta, Indonesia; 9 Centre for Tropical Medicine and Global Health, Nuffield Department of Clinical Medicine, University of Oxford, Oxford, United Kingdom; Johns Hopkins Bloomberg School of Public Health, UNITED STATES

## Abstract

**Background:**

*Plasmodium malariae* is a slow-growing parasite with a wide geographic distribution. Although generally regarded as a benign cause of malaria, it has been associated with nephrotic syndrome, particularly in young children, and can persist in the host for years. Morbidity associated with *P*. *malariae* infection has received relatively little attention, and the risk of *P*. *malariae*-associated nephrotic syndrome is unknown.

**Methodology/Principal Findings:**

We used data from a very large hospital-based surveillance system incorporating information on clinical diagnoses, blood cell parameters and treatment to describe the demographic distribution, morbidity and mortality associated with *P*. *malariae* infection in southern Papua, Indonesia. Between April 2004 and December 2013 there were 1,054,674 patient presentations to Mitra Masyarakat Hospital of which 196,380 (18.6%) were associated with malaria and 5,097 were with *P*. *malariae* infection (constituting 2.6% of all malaria cases). The proportion of malaria cases attributable to *P*. *malariae* increased with age from 0.9% for patients under one year old to 3.1% for patients older than 15 years. Overall, 8.5% of patients with *P*. *malariae* infection required admission to hospital and the median length of stay for these patients was 2.5 days (Interquartile Range: 2.0–4.0 days). Patients with *P*. *malariae* infection had a lower mean hemoglobin concentration (9.0g/dL) than patients with *P*. *falciparum* (9.5g/dL), *P*. *vivax* (9.6g/dL) and mixed species infections (9.3g/dL). There were four cases of nephrotic syndrome recorded in patients with *P*. *malariae* infection, three of which were in children younger than 5 years old, giving a risk in this age group of 0.47% (95% Confidence Interval; 0.10% to 1.4%). Overall, 2.4% (n = 16) of patients hospitalized with *P*. *malariae* infection subsequently died in hospital, similar to the proportions for the other endemic *Plasmodium* species (range: 0% for *P*. *ovale* to 1.6% for *P*. *falciparum*).

**Conclusions/Significance:**

*Plasmodium malariae* infection is relatively uncommon in Papua, Indonesia but is associated with significant morbidity from anemia and a similar risk of mortality to patients hospitalized with *P*. *falciparum* and *P*. *vivax* infection. In our large hospital database, one in 200 children under the age of 5 years with *P*. *malariae* infection were recorded as having nephrotic syndrome.

## Author Summary

*Plasmodium malariae* is a relatively rare, but widely distributed, cause of malaria. It can persist in the human host for years, often without causing significant symptoms. As a result, *P*. *malariae* will be a very difficult species to eradicate. Our study used data from a routine hospital-based surveillance system in southern Papua, Indonesia to describe the clinical epidemiology of *P*. *malariae* infections. Over a 10-year period there were 5,097 patient presentations to Mitra Masyarakat Hospital associated with *P*. *malariae* infection constituting 2.6% of all malaria cases. Patients with *P*. *malariae* malaria had a significantly older age distribution than those with *P*. *vivax* infections. They also had lower mean hemoglobin concentrations than patients infected with *P*. *falciparum*, *P*. *vivax* or mixed *Plasmodium* species. We speculate that this may be due to chronic hemolysis of parasitized and non-parasitized red cells as a result of persistent infection. One in 200 children under the age of 5 years with *P*. *malariae* infection were recorded as having nephrotic syndrome, a well-known but to date unquantified complication. Overall, 0.3% of patients with *P*. *malariae* malaria died. These findings emphasize the need to consider this parasite when designing comprehensive malaria elimination strategies.

## Introduction

*Plasmodium malariae* is one of the five *Plasmodium* species that commonly infect humans. The global incidence of infection by this species is unknown but is thought to be significantly lower than for *P*. *falciparum* [1]. *Plasmodium malariae* is endemic in parts of Africa [2–4], South America [5, 6], Asia [7–10] and the Western Pacific [11]. Infection is often asymptomatic [12] and severe disease is thought to be rare. However, untreated infection has been reported to lead to nephrotic syndrome [13–15] and albuminuria was commonly noted in patients treated with *P*. *malariae* for neurosyphilis in the 1930s [16]. Given the parasite’s ability to survive in the human host at low parasitemias for decades [17], chronic morbidity related to *P*. *malariae* infection is likely to occur.

Global malaria elimination strategies justifiably target *P*. *falciparum*, which is associated with the greatest risk of acute morbidity and mortality. When such malaria control strategies are successful, the fall in *P*. *falciparum* endemicity is often associated with a relative rise in the burden of malarial disease caused by the non-falciparum malarias [18, 19]. This study was conducted to investigate the demographic distribution, morbidity and mortality associated with *P*. *malariae* infection in southern Papua, Indonesia, a malarious region coendemic for four *Plasmodium* species–*P*. *falciparum*, *P*. *vivax*, *P*. *malariae* and *P*. *ovale*. Understanding the features of *P*. *malariae* infection will be important for guiding clinical management and eventual eradication of this species.

## Methods

### Study site

This study was conducted at Rumah Sakit Mitra Masyarakat (RSMM), the major referral hospital in Timika, southcentral Papua, Indonesia. The characteristics of this hospital and the surrounding region have been described in detail elsewhere [20, 21]. In brief, Timika, has a population of approximately 200,000 people and is situated in the lowlands, about 50km south of a large copper and gold mine. It has a tropical climate with rainfall occurring year-round with minimal seasonal variation. Timika has a diverse ethnic population comprised of Highland Papuans, Lowland Papuans (both of Melanesian descent) and non-Papuans (mostly of Indonesian descent). Rumah Sakit Mitra Masyarakat has 110 beds, a 24h emergency department, a high dependency unit with facilities for intravenous infusions, peritoneal dialysis and monitoring but not mechanical ventilation, and an outpatient department that sees approximately 300 patients per day, 6 days per week.

Papua has the highest burden of malaria in Indonesia with an estimated incidence in Timika of 876 episodes per 1,000 per year (range: 711–906) [20]. High frequencies of drug resistant *P*. *falciparum* and *P*. *vivax* strains have been reported [22] although *P*. *malariae* remains sensitive to chloroquine [23]. Malaria transmission is perennial and most intense in the lowland regions. In view of the high levels of antimalarial drug resistance exhibited by both *P*. *falciparum* and *P*. *vivax*, antimalarial policy for uncomplicated malaria due to any *Plasmodium* species was changed in March 2006 from oral quinine to dihydroartemisinin-piperaquine, an artemisinin combination therapy (ACT). In order to monitor the impact of ACT on malaria morbidity and mortality, a hospital surveillance system was established in 2004 and maintained until 2013.

### Data collection procedures

Data from all patient presentations to RSMM between April 2004 and December 2013 were eligible for inclusion in this study. Hospital administrators entered demographic and diagnostic data into an electronic database (Q-Pro software, Jakarta, Indonesia) for all patients presenting to the hospital, regardless of department. Data collected included age, gender, ethnicity, pregnancy status and diagnoses classified according to the International Classification of Diseases, version 10. The latter was based on the opinion of the treating physician after clinical investigation. The results of full blood counts from the hospital’s Coulter Counter were entered automatically into a separate database and prescription data from the hospital pharmacy were entered manually into a further database by the pharmacist filling the prescription. Records from all three databases were identified using the patient’s unique hospital identification number. Previous published analyses have included data from patients presenting to RSMM between 2004 and the end of 2012 [24, 25].

Hospital guidelines dictated that all febrile patients seen in the outpatients department and all inpatients regardless of clinical diagnosis had a blood film for malaria. Giemsa-stained thick and thin films were considered negative after microscopic assessment of 100 high power fields. Hospital microscopists underwent regular training and quality assurance procedures to ensure a high standard of microscopy [21].

Clinical, hematological and prescription data were merged sequentially by creating all possible pairwise combinations for a given unique hospital identification number. Sets where the date of the laboratory record or prescription fell between the date of admission and discharge were retained. In cases where there was more than one hemoglobin or platelet count during a single presentation, only the lowest value was kept. Both the minimum and the maximum white cell count were kept. Severe anemia was defined as a hemoglobin less than 5g/dL but in accordance with 2014 World Health Organization severe malarial anemia definitions, we also conducted subanalyses in which severe anemia was defined as a hemoglobin <5g/dL in children under 12 years of age and <7g/dL in adults ≥12 years of age [26]. Severe thrombocytopenia was defined as a platelet count less than 50x10^3^/μL. White cell counts were categorized as normal or abnormal according to age-specific normal ranges [27].

### Statistical analysis

Analyses were performed using STATA version 13.1 (College Station, Texas, USA). Anemia, thrombocytopenia, admission to hospital and length of stay were used to explore the morbidity associated with *P*. *malariae* infection and were compared between patients infected with *P*. *falciparum*, *P*. *vivax*, *P*. *ovale* (where numbers allowed) and mixed *Plasmodium* species infections. We also examined the frequency of abnormal white cell counts and selected comorbidities to help establish whether *P*. *malariae* infection was likely to have been the sole reason for presentation. Univariable and multivariable logistic regression models (the latter adjusting for age group (<1 year, 1 to <5 years, 5 to <15 years, ≥15 years), sex, ethnicity (non-Papuan, Highland Papuan, Lowland Papuan), pregnancy status and white cell count (normal, abnormal) were used to compare the odds for severe anemia and severe thrombocytopenia between patients with *P*. *malariae* infection and those with infection by the other *Plasmodium* species listed above. As individuals in the hospital database could appear more than once, robust standard errors (Huber-White sandwich estimator) were calculated, accounting for within-patient correlation. Given the very large numbers of patients in the database, formal tests of statistical significance were only done in the context of univariable and multivariable logistic regression models.

### Ethical clearance

This study was approved by the Ethics committees of the University of Gajah Mada (Yogyakarta, Indonesia) and Menzies School of Health Research (Darwin, Australia). All data were anonymized.

## Results

Between April 2004 and December 2013 there were 1,054,674 presentations to RSMM of which 196,380 (18.6%) were associated with malaria. *Plasmodium malariae* monoinfection accounted for 2.6% of all malaria cases (5,097 presentations made by 4,456 individuals) (Table 1). Other *Plasmodium* species present included *P*. *falciparum* (100,078 presentations, 51.0%), *P*. *vivax* (65,306 presentations, 33.3%), *P*. *ovale* (120 presentations, 0.1%) and mixed *Plasmodium* species (25,779 presentations, 13.1%). Two hundred and forty one (0.9%) of the mixed species infections included *P*. *malariae*, of which 148 (0.6%) were in combination with *P*. *falciparum* and 93 (0.4%) were with *P*. *vivax*. Overall, 4.4% (225/5,097) of presentations with *P*. *malariae* monoinfection were followed by a further presentation with *P*. *malariae* infection within one year. The median day of representation was 171 (Interquartile Range [IQR] 86–266) with no significant difference in the crude risk between those treated with oral quinine and those treated with dihydroartemisinin-piperaquine (5.9% versus 4.4%). Only 1.3% (10,886/858,294) of patients without malaria, 1.9% (1,892/100,078) of patients with falciparum malaria and 1.3% (868/65,306) of patients with vivax malaria represented with *P*. *malariae* infection within a year.

**Table 1:** Demographic distribution of all 1,054,674 patient presentations.

	Species						
	Negative	*P*. *falciparum*	*P*. *vivax*	*P*. *ovale*	*P*. *malariae*	Mixed species	Total
	n (%)	n (%)	n (%)	n (%)	n (%)	n (%)	n (%)
**Age group**							
0 to <1	71,789 (8.4)	2,321 (2.3)	5,274 (8.1)	2 (1.7)	80 (1.6)	992 (3.8)	80,458 (7.6)
1 to <5	116,487 (13.6)	16,132 (16.1)	19,859 (30.4)	11 (9.2)	554 (10.9)	6,152 (23.9)	159,195 (15.1)
5 to <15	86,366 (10.1)	19,913 (19.9)	11,340 (17.4)	22 (18.3)	1,096 (21.5)	5,559 (21.6)	124,296 (11.8)
≥15	583,607 (68.0)	61,707 (61.7)	28,832 (44.1)	85 (70.8)	3,367 (66.1)	13,075 (50.7)	690,673 (65.5)
**Sex**							
Male	378,074 (44.0)	49,592 (49.6)	32,970 (50.5)	68 (56.7)	2,575 (50.5)	13,068 (50.7)	476,347 (45.2)
Female	480,220 (56.0)	50,486 (50.4)	32,336 (49.5)	52 (43.3)	2,522 (49.5)	12,711 (49.3)	578,327 (54.8)
**Pregnant**							
No	399,336 (83.2)	47,808 (94.7)	31,215 (96.5)	49 (94.2)	2,395 (95.0)	12,264 (96.5)	493,067 (85.3)
Yes	80,884 (16.8)	2,678 (5.3)	1,121 (3.5)	3 (5.8)	127 (5.0)	447 (3.5)	85,260 (14.7)
**Ethnic group**							
Non-Papuan	152,502 (17.8)	8,305 (8.3)	7,539 (11.6)	9 (7.5)	164 (3.2)	1,324 (5.1)	169,843 (16.1)
Highland Papuan	576,524 (67.3)	81,706 (81.7)	52,905 (81.1)	106 (88.3)	4,452 (87.3)	22,434 (87.0)	738,127 (70.1)
Lowland Papuan	128,027 (14.9)	9,982 (10)	4,820 (7.4)	5 (4.2)	481 (9.4)	2,015 (7.8)	145,330 (13.8)
**Total**	858,294 (100)	100,078 (100)	65,306 (100)	120 (100)	5,097 (100)	25,779 (100)	1,054,674 (100)

Abbreviations: n, number

Age data were missing in 52 cases, and ethnicity data were missing in 1,374 cases

**Fig 1:**
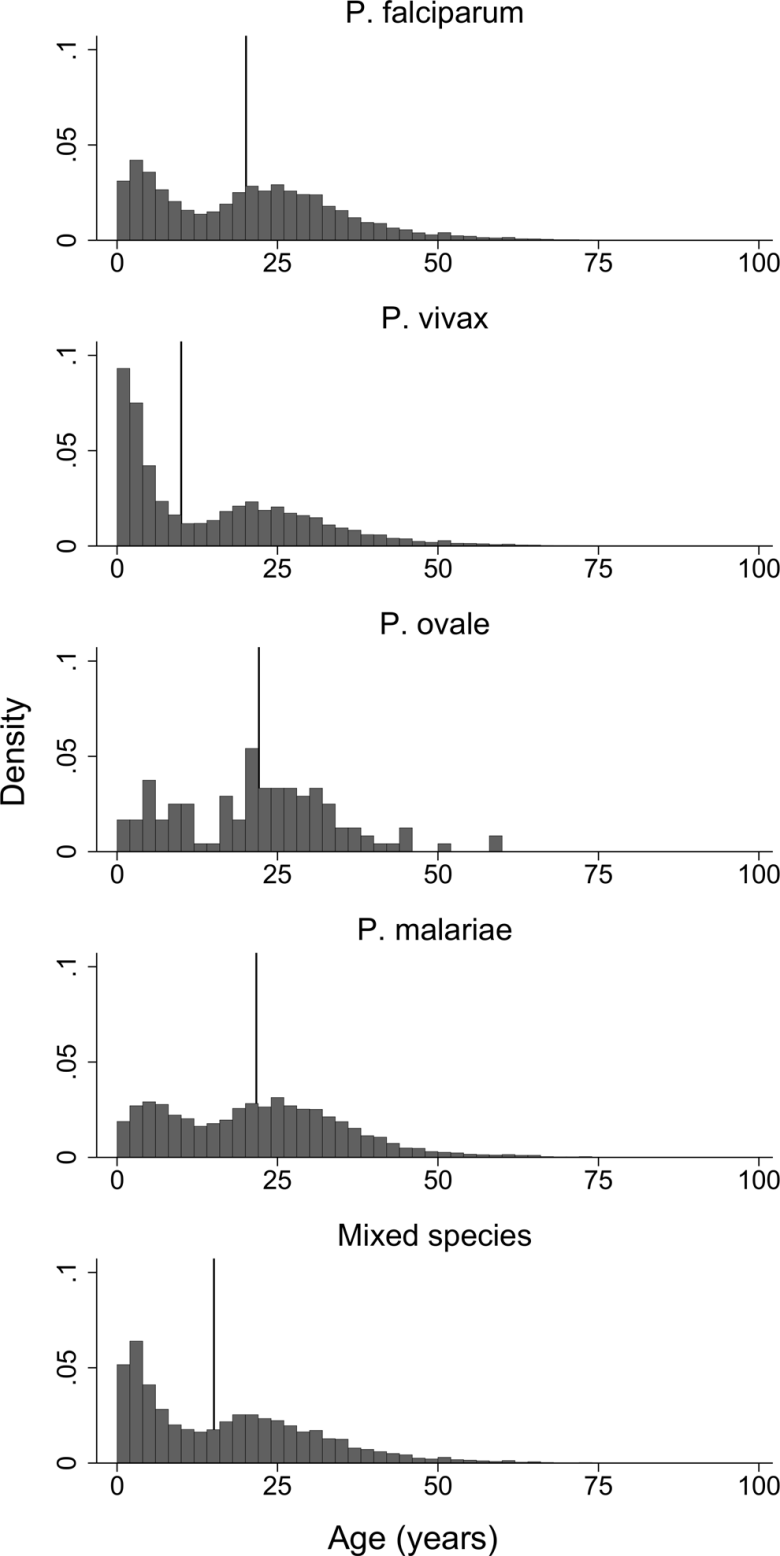
Age distributions of patients presenting to RSMM by *Plasmodium* species. Vertical lines represent median age.

**Fig 2:**
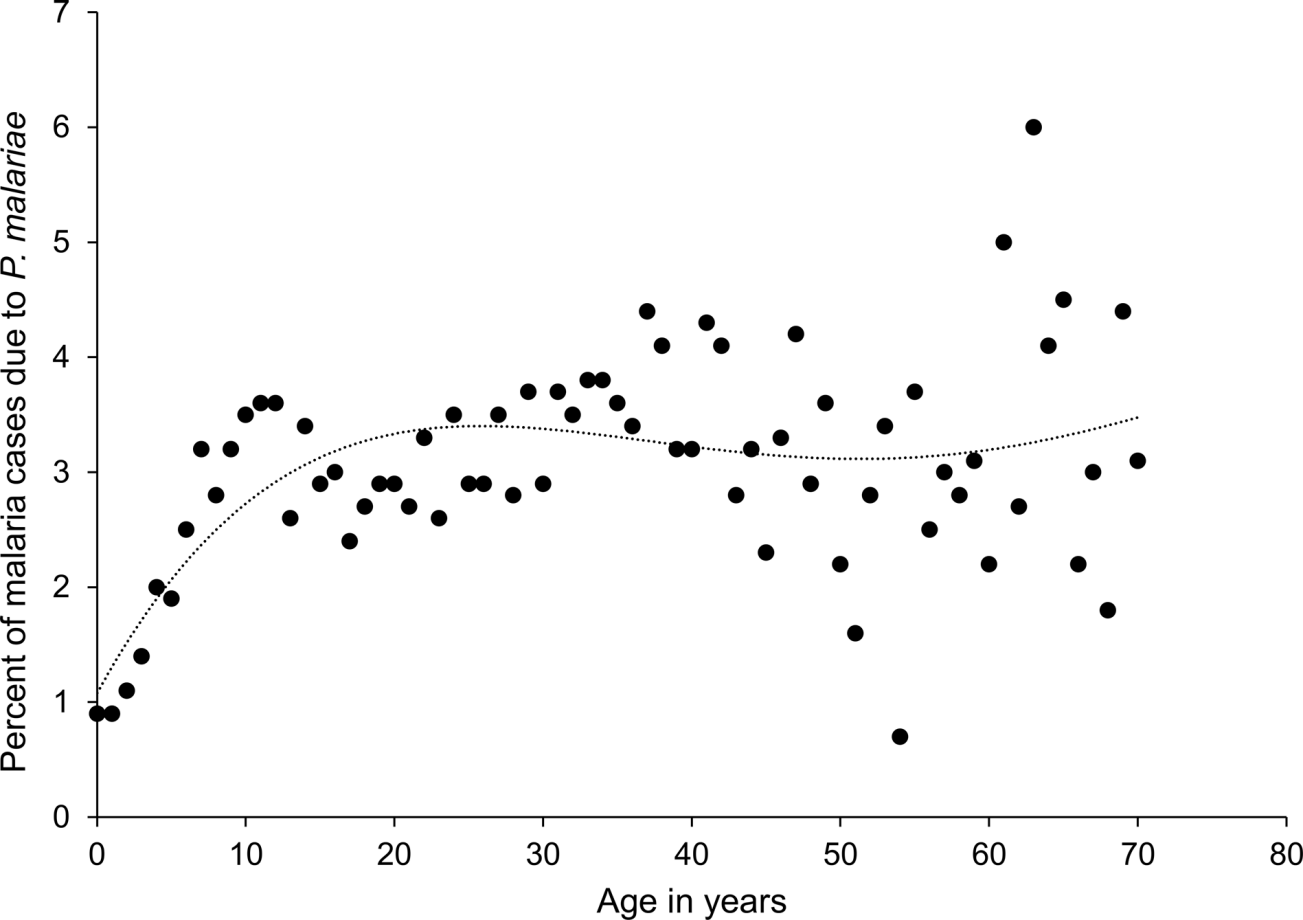
Percent of malaria cases due to *Plasmodium malariae* by age, with polynomial fit line. Age truncated at 70 years to maintain stability of fit line.

The age distribution of all patients presenting with *P*. *malariae* monoinfection was similar to *P*. *falciparum* (median 21.7 and 20.1 years respectively), but substantially older than for *P*. *vivax (*median 10.0 years) and mixed infections (15.1 years). For all species, there was a bimodal distribution of age at presentation with a peak in children under 5 years of age and another in early adulthood. The childhood peak for *P*. *malariae* was less pronounced than for the other species (*P*. *ovale* excepted) (see Fig 1). The proportion of malaria presentations attributable to *P*. *malariae* increased from 0.9% (80/8,669) for patients less than one year of age to 3.1% (3,367/107,066) for patients older than 15 years (Fig 2).

The majority of presentations with *P*. *malariae* were in Highland Papuans (4,452, 87.3%), but this predominance was also apparent for the other *Plasmodium* species (range 81.0–88.3%) and patients without malaria (67.2%).

### Hospitalization, treatment and comorbidity

In total, 8.5% (432/5,097) of the patients with *P*. *malariae* monoinfection required admission to hospital, less than the proportion for patients with *P*. *falciparum* (17.4%) and mixed infections (15.3%), but similar to those with *P*. *vivax* (9.4%) and *P*. *ovale* (8.3%) infections. The median length of stay for those admitted with *P*. *malariae* infection was 2.5 days (IQR 2.0–4.0 days); shorter than for those without malaria 3.0 days (IQR 2.0–5.0 days) but longer than for patients with other *Plasmodium* species infections (*P*. *falciparum*, 2.0 days (2.0–4.0), *P*. *vivax*, 2.0 days (2.0–4.0), *P*. *ovale*, 2.0 days (1.0–3.0) and mixed species, 2.0 days (2.0–4.0). Admission for greater than 5 days was required for 17.4% (75/432) of patients with *P*. *malariae* infection, 19.4% (13,130/67,365) of those without malaria, 11.2% (1,942/17,387) with *P*. *falciparum*, 13.0% (795/6,116) with *P*. *vivax*, 10.0% (1/10) with *P*. *ovale* and 12.0% (473/3,945) with mixed species infections. Assuming 100% bed occupancy over the study period, patients with *P*. *malariae* infection accounted for 0.4% (1,733/391,710) of total hospital bed occupancy. The corresponding figures for the other *Plasmodium* species were 14.6% (57,037 bed days) for *P*. *falciparum*, 5.4% (21,298 bed days) for *P*. *vivax*, 0.006% (24 bed days) for *P*. *ovale* and 3.4% (13,241 bed days) for mixed species infections.

Overall, 4,691 (92.0%) patient presentations with *P*. *malariae* infection were matched with corresponding pharmacy data. In 4,459 of these cases (95.1%), treatment was with oral therapy alone. Prior to March 2006 (401 cases), the majority of patients received either oral quinine (n = 212; 52.9%), chloroquine (n = 76; 19.0%) or doxycycline (n = 71, 17.7%) with small numbers receiving sulphadoxine-pyrimethamine (n = 21, 5.2%). After the change of malaria treatment protocols to ACT in March 2006 (4,290 cases), 4,157 (96.9%) were treated with ACT (3,898 (90.9%) with dihydroartemisinin-piperaquine and 261 (6.1%) with artesunate-amodiaquine (two patients received both combinations)). In 232 cases (4.9%), patients with *P*. *malariae* were treated with parenteral therapy; intravenous quinine prior to April 2006 (23 cases, 9.9%) and intravenous artesunate after this date (209 cases, 90.1%).

Of the 5,097 presentations with *P*. *malariae* monoinfection, 1,516 (29.7%) were associated with at least one additional clinical diagnosis and this figure rose to 96.5% (417 presentations) in those admitted to hospital. The overall proportions with comorbidity for those with *P*. *falciparum*, *P*. *vivax* and mixed infections were slightly higher: 32.7%, 30.5% and 31.0% respectively. Thirty (0.6%) patients with *P*. *malariae* infection presented following trauma and therefore presumably had incidental parasitemias (Table 2). Overall, 16 (0.3%) of the patients with *P*. *malariae* infection had documented renal disease, of whom 4 (0.1%) were recorded as having nephrotic syndrome. Although numbers were small, this proportion was approximately 10-fold higher than for the other *Plasmodium* species (Table 2). All four of the patients with *P*. *malariae* infection and nephrotic syndrome were under the age of 15 years and three were under the age of 5 years giving a risk of nephrotic syndrome of 0.23% (95% Confidence Interval [95%CI]; 0.06% to 0.59%) in children under 15 years and 0.47% (95%CI; 0.10% to 1.4%) in children aged under 5 years old. None of the 4 patients with nephrotic syndrome died at the hospital prior to the end of the study.

**Table 2:** Comorbidities in patients presenting to Rumah Sakit Mitra Masyarakat by *Plasmodium* species.

	Species						
	Negative	*P*. *falciparum*	*P*. *vivax*	*P*. *ovale*	*P*. *malariae*	Mixed species	Total
	n (%)	n (%)	n (%)	n (%)	n (%)	n (%)	n (%)
**Pneumonia**							
No	834,991 (97.3)	98,983 (98.9)	64,368 (98.6)	119 (99.2)	5,053 (99.1)	25,436 (98.7)	1,028,950 (97.6)
Yes	23,303 (2.7)	1,095 (1.1)	938 (1.4)	1 (0.8)	44 (0.9)	343 (1.3)	25,724 (2.4)
**Renal Disease**							
No	855,722 (99.7)	99,663 (99.6)	65,225 (99.9)	120 (100)	5,081 (99.7)	25,695 (99.7)	1,051,506 (99.7)
Yes	2,572 (0.3)	415 (0.4)	81 (0.1)	0 (0)	16 (0.3)	84 (0.3)	3,168 (0.3)
**Nephrotic syndrome**							
No	858,117 (99.98)	100,072 (99.99)	65,302 (99.99)	120 (100)	5,093 (99.9)	25,776 (99.99)	1,054,480 (99.98)
Yes	177 (0.02)	6 (0.01)	4 (0.01)	0 (0)	4 (0.1)	3 (0.01)	194 (0.02)
**HIV**							
No	840,095 (97.9)	99,498 (99.4)	65,029 (99.6)	119 (99.2)	5,072 (99.5)	25,661 (99.5)	1,035,474 (98.2)
Yes	18,199 (2.1)	580 (0.6)	277 (0.4)	1 (0.8)	25 (0.5)	118 (0.5)	19,200 (1.8)
**Trauma**							
No	800,208 (93.2)	99,664 (99.6)	65,046 (99.6)	120 (100)	5,067 (99.4)	25,708 (99.7)	995,813 (94.4)
Yes	58,086 (6.8)	414 (0.4)	260 (0.4)	0 (0)	30 (0.6)	71 (0.3)	58,861 (5.6)

Abbreviations: n; number, HIV; human immunodeficiency virus

### Hematological morbidity

Hemoglobin concentrations, platelet counts and white cell counts were available in 22.9% (241,594), 22.4% (236,536) and 22.7% (239,444) of all patient presentations with the corresponding figures for *P*. *malariae* cases being 34.3% (1,750), 33.6% (1,713) and 34.1% (1,739) respectively. The mean hemoglobin concentration was 9.0 g/dL in patients with *P*. *malariae* infection, the lowest of all the *Plasmodium* species (10.6g/dL for those without malaria, 9.5g/dL for *P*. *falciparum*, 9.6g/dL for *P*. *vivax*, and 9.3g/dL for mixed species infections). Overall, 5.7% (n = 100) of patients presenting with *P*. *malariae* infections had a hemoglobin <5g/dL (32/577 [5.5%] of those <12 years and 68/1,173 [5.8%] of those ≥12 years). This was similar to the crude proportions for *P*. *falciparum* and *P*. *vivax* (Table 3). Three hundred and sixty nine (21.1%) patients with *P*. *malariae* malaria had hemoglobin concentrations under 7g/dL (151/577 [26.2%] of those <12 years and 218/1,173 (18.6%) of those ≥12 years) and 1,128 (64.5%) had a hemoglobin under 10g/dL. After controlling for confounding factors, those with *P*. *malariae* infection were at significantly greater risk of severe anemia compared to those without *Plasmodium* infection both when defined as a hemoglobin of less than 5g/dL (Adjusted Odds Ratio [AOR]; 2.29, 95%CI; 1.85–2.84, *P*<0.001, Table 4) and as per 2014 WHO criteria (AOR; 2.02, 95%CI; 1.75–2.33, *P*<0.001) [26].

**Table 3:** Proportions with severe anemia (hemoglobin <5g/dL), severe thrombocytopenia (platelet count <50 x10^3^/μL) and abnormal white cell count by *Plasmodium* species.

	Severe anemia			Severe thrombocytopenia		White cell count	
Species	Hb ≥5g/dL, n (%)	Hb <5g/dL, n (%)	WHO criteria*, n (%)	Plt ≥50x10^3^/μL, n (%)	Plt <50 x10^3^/μL, n (%)	Normal, n (%)	Abnormal, n (%)
Negative	163,650 (97.5)	4,136 (2.5)	12,051 (7.2)	159,755 (97.3)	4,417 (2.7)	94,929 (57.1)	71,224 (42.9)
*P*. *malariae*	1,650 (94.3)	100 (5.7)	250 (14.3)	1,590 (92.8)	123 (7.2)	1,141 (65.6)	598 (34.4)
*P*. *falciparum*	37,585 (93.7)	2,521 (6.3)	4,851 (12.1)	33,353 (85.0)	5,905 (15.0)	26,956 (67.8)	12,812 (32.2)
*P*. *vivax*	21,085 (95.0)	1,099 (5.0)	1,805 (8.1)	20,044 (92.0)	1,743 (8.0)	15,422 (69.9)	6,644 (30.1)
*P*. *ovale*	31 (100)	0 (0)	3 (9.7)	28 (93.3)	2 (6.7)	21 (72.4)	8 (27.6)
Mixed species	8,955 (92.0)	782 (8.0)	1,239 (12.7)	8,345 (87.1)	1,231 (12.9)	6,674 (68.9)	3,015 (31.1)
**Total**	232,956 (96.4)	8,638 (3.6)	20,199 (8.4)	223,115 (94.3)	13,421 (5.7)	145,143 (60.6)	94,301 (39.4)

Abbreviations: n; number, WHO; World Health Organization, Hb; hemoglobin, Plt; platelet

* Hemoglobin < 5g/dL in children under 12 years old and <7g/dL in adults ≥12 years old

**Table 4:** Univariable and multivariable logistic regression models of the risk factors for severe anemia (hemoglobin <5g/dL) and severe thrombocytopenia (platelet count <50 x10^3^/μL).

Severe anemia (Hb<5g/dL)					Severe thrombocytopenia (Plt<50x10^3^/μL)			
Risk factor	Crude OR (95%CI)	*P*	AOR^a^ (95%CI)	*P*	Crude OR (95%CI)	*P*	AOR^a^ (95%CI)	*P*
**Species**								
Negative	ref	-	ref	-	ref	-	ref	-
*P*. *malariae*	2.40 (1.94–2.96)	<0.001	2.29 (1.85–2.84)	<0.001	2.80 (2.32–3.38)	<0.001	2.34 (1.93–2.83)	<0.001
*P*. *falciparum*	2.65 (2.51–2.81)	<0.001	2.24 (2.11–2.38)	<0.001	6.40 (6.07–6.76)	<0.001	6.20 (5.86–6.55)	<0.001
*P*. *vivax*	2.06 (1.92–2.22)	<0.001	2.03 (1.89–2.19)	<0.001	3.15 (2.94–3.37)	<0.001	3.88 (3.62–4.15)	<0.001
Mixed species	3.46 (3.18–3.76)	<0.001	3.58 (3.28–3.91)	<0.001	5.34 (4.95–5.75)	<0.001	5.86 (5.42–6.34)	<0.001
**Age group**								
<1 year	0.98 (0.89–1.08)	0.6	1.02 (0.92–1.12)	0.8	0.44 (0.41–0.48)	<0.001	0.47 (0.43–0.52)	<0.001
1 to <5 years	1.58 (1.48–1.69)	<0.001	1.25 (1.16–1.34)	<0.001	0.46 (0.43–0.49)	<0.001	0.31 (0.28–0.33)	<0.001
5 to <15 years	1.49 (1.37–1.61)	<0.001	1.15 (1.06–1.25)	0.001	0.80 (0.75–0.85)	<0.001	0.48 (0.45–0.51)	<0.001
≥15 years	ref	-	ref	-	ref	-	ref	-
**Gender**								
Male	ref	-	ref	-	ref	-	ref	-
Female	1.05 (0.99–1.11)	0.1	-^b^	-	0.76 (0.72–0.79)	<0.001	- ^b^	-
**Pregnancy status**								
Not pregnant	ref	-	1.17 (1.10–1.24) ^b^	<0.001	ref	-	0.75 (0.71–0.79)^b^	<0.001
Pregnant	0.62 (0.57–0.68)	<0.001	0.99 (0.89–1.10) ^b^	0.9	0.65 (0.60–0.71)	<0.001	0.61 (0.55–0.66)^b^	<0.001
**Ethnic Group**								
Non-Papuan	ref	-	ref	-	ref	-	ref	-
Highland Papuan	4.22 (3.75–4.74)	<0.001	3.89 (3.45–4.38)	<0.001	3.06 (2.83–3.30)	<0.001	2.89 (2.66–3.14)	<0.001
Lowland Papuan	2.64 (2.30–3.04)	<0.001	2.52 (2.18–2.91)	<0.001	0.87 (0.78–0.97)	0.02	0.99 (0.89–1.12)	0.9
**White cell count**								
Normal	ref	-	ref	-	ref	-	ref	-
Abnormal	1.85 (1.77–1.94)	<0.001	2.07 (1.97–2.18)	<0.001	1.34 (1.29–1.39)	<0.001	1.77 (1.70–1.85)	<0.001

Abbreviations: OR; odds ratio, AOR; adjusted odds ratio, Hb; hemoglobin, Plt; platelet

^a^ Multivariable models also include year of presentation

^b^ In the multivariable model, a non-ordered, 3-category variable incorporating sex and pregnancy status was used. Male sex is the reference category.

The mean platelet count in patients with *P*. *malariae* infection was 142.7x10^3^/μL, compared to 130.4x10^3^/μL for *P*. *falciparum*, 166.8x10^3^/μL for *P*. *vivax* and 131.6x10^3^/μL for mixed species infection. Severe thrombocytopenia (platelet count <50x10^3^/μL) was present in 123 (7.2%) patients with *P*. *malariae* (Table 3) and very severe thrombocytopenia (platelet count <20x10^3^/μL) in 16 (0.9%) cases. None of the patients with platelet counts <50x10^3^/μL) were recorded as having clinical evidence of bleeding. After controlling for confounding factors, *P*. *malariae* infection was associated with a two-fold higher odds of severe thrombocytopenia compared to those with no malaria (AOR; 2.34, 95%CI; 1.93–2.83, *P*<0.001) (Table 4).

Overall 34.4% (n = 598) of patients with *P*. *malariae* infection had an abnormal age-adjusted white cell count; a similar proportion to patients infected with the other *Plasmodium* species, but lower than that in patients without malaria (42.9%, *P*<0.001). In 36.8% of cases the abnormal white cell count was above the age-adjusted normal range and in the remaining 63.2% it was below the normal range.

### Mortality

In total, 0.3% (16/5,097) of patients with *P*. *malariae* infection died compared to 0.5% (4,254/858,294) of those without malaria, 0.4% (376/100,078) with *P*. *falciparum*, 0.2% (130/65,306) with *P*. *vivax*, none with *P*. *ovale* and 0.3% (73/25,779) with mixed infections. The corresponding risk of mortality for patients who were admitted to hospital or seen in the emergency department was 2.4% (16/663) for *P*. *malariae*, 3.4% (4,244/125,375) for those without *Plasmodium* infection, 1.6% (375/23,304) for *P*. *falciparum*, 1.4% (129/8,937) for *P*. *vivax* and 1.6% (73/4,608) for mixed infections. The median age of the 16 patients who died with *P*. *malariae* parasitemia was 23.4 years (Range; 5.6 to 50 years, IQR; 17.0–41.9 years), with a risk of mortality of 1/1,481 (0.07%) in children <12 years and 15/3,616 (0.4%) in adults ≥12 years. Nine (56.3%) of the patients who died were male, 4 (26.7%) had a hemoglobin <5g/dL and 6 (40%) had severe anemia according to 2014 WHO criteria (Table 5). Two patients (14.3%) had severe thrombocytopenia and 10 (71.4%) had an abnormal white cell count (9 patients (90%) had a value that was above the normal range during their admission and three (30%) had a value that was below the normal range). Five (31%) patients were recorded as having concomitant tuberculosis.

**Table 5:** Hematological parameters and recorded comorbidities in the 16 patients who died with *Plasmodium malariae* parasitemia.

Patient Number	Sex	Age (years)	Length of stay (days)	Minimum hemoglobin concentration (g/dL)	Platelet count (x10^3^/μL)	Most abnormal white cell count (cells/μL)	Other recorded diagnoses
1.	M	33.7	6	2.7	146	6,100	Ischaemic cardiomyopathy
							Congestive heart failure
							Non-inflammatory pericardial effusion
							Pneumonia
2.	M	22.6	13	9.6	182	2,600	HIV
							Tuberculous meningitis
							Gastroenteritis
3.	M	37.6	0.5	11.1	343	16,800	Pulmonary tuberculosis
4.	M	21.3	9	3.1	53	13,100	Acute renal failure
5.	F	12.2	3	7.8	199	29,000	Malnutrition
							Helminthiasis
							Pulmonary tuberculosis
6.	F	18	0.5	-	-	-	-
7.	F	18.7	1	11.9	93	10,500	Pulmonary tuberculosis
							Gastroenteritis
							Urinary tract infection
8.	F	12	0.5	3.3	-	15,600	-
9.	M	49.1	0.5	6.7	23	14,700	Pneumonia
							Non-infective gastroenteritis
							Chronic renal failure
10.	F	46.1	1	11	44	4,100	Pulmonary tuberculosis
							Gastroenteritis
11.	F	16	6	4.8	339	13,200	Chronic renal failure
12.	M	24.1	0	7.2	140	6,000	-
							Pneumonia
							Adult Respiratory Distress Syndrome
14.	M	50	1	6.9	128	8,300	Pneumonia
							Chronic renal failure
							Suspected tuberculosis
							Inguinal hernia
							Ascites
15.	M	5.6	3	11.1	56	3,500	Encephalomyelitis
16.	F	48	0	10.6	156	-	-

Abbreviations: F = female, M = male

## Discussion

This large case series from southern Papua shows that *P*. *malariae* is responsible for a minority (2.6%) of clinical malaria cases but has potential to cause significant morbidity. Compared to patients infected with other *Plasmodium* species, those with *P*. *malariae* infection had a lower mean hemoglobin concentration and a similar risk of being admitted to hospital (8.5%) or dying (0.3%). Overall *P*. *malariae* malaria accounted for almost 0.5% of hospital bed occupancy.

Patients with *P*. *malariae* infections tended to be older than those with non *P*. *malariae* malaria, particularly compared to those with *P*. *vivax* mono- or mixed infections. This probably reflects the low transmission intensity in this area. Whereas the majority of children in Timika are likely to have been infected with *P*. *vivax* by the age of 5 years (and therefore have started to acquire immunity early in life), fewer than one in ten children could be expected to have been infected with *P*. *malariae* by the same age (based on an estimated annual incidence of 15.7 cases per 1,000 persons in 2005) [20]. *Plasmodium malariae* has a predilection for senescent red blood cells whereas *P*. *vivax* preferentially infects young red cells, in particular reticulocytes [28, 29]. It is therefore possible that during the very early stages of infancy there is a biological predisposition to vivax malaria and a relative protection against *P*. *malariae* infection.

A major finding of our study (and one that was also noted in an earlier published analysis from this region [24]) is the association between *P*. *malariae* parasitemia and substantial hematological morbidity. The mean hemoglobin in patients with *P*. *malariae* infection was 0.3–0.6g/dL lower than the other locally prevalent *Plasmodium* species with an associated 2.3-fold greater odds of severe anemia compared to individuals without malaria. There are several potential explanations for this finding. *Plasmodium malariae* replicates slowly and does not typically reach high parasitemias [29]. Prolonged infection is therefore more likely to have been present in patients presenting with this disease compared to those with *P*. *falciparum* or *P*. *vivax* infections. A chronic, low-level parasitemia resulting in ongoing destruction of both parasitized but, more importantly, non-parasitized red cells as well as marrow dyserythropoiesis may have a cumulative effect on lowering hemoglobin concentrations. Previous observation of malariatherapy patients treated with *P*. *malariae* infection demonstrated that those with naturally-induced infections typically had a nadir in hemoglobin concentration between day 30 and 90 of infection with only a slight improvement in concentrations thereafter [16]. Recent data suggest that the low circulating parasitemias found in *P*. *vivax* infection significantly underestimate total parasite biomass [30]. While speculative, it is possible that a hidden, non-circulating parasite biomass may also be present in *P*. *malariae*, capable of contributing to a degree of anemia out of proportion to circulating parasitemia.

It is also possible that the significant anemia seen with *P*. *malariae* infection is partially a result of comorbid conditions as opposed to the infection itself. However, given the similar frequency of major comorbidities such as pneumonia, renal disease and HIV compared with the other species, a major bias from differential rates of comorbidities seems unlikely. Finally, Highland Papuans are the ethnic group with the highest risk of severe anemia in Timika–possibly due to nutritional factors, red cell and hemoglobin abnormalities or gastrointestinal helminth infection. Whether for geographical or biological reasons, a particularly high proportion of *P*. *malariae* patients were highlanders, potentially contributing to the lower mean hemoglobin seen in these patients. After adjusting for various risk factors, including ethnicity, in the multivariable model, the risk of severe anemia in *P*. *malariae* infections was similar to that for *P*. *falciparum* and *P*. *vivax* monoinfections.

The low incidence of *P*. *malariae* infection and relatively high burden of falciparum and vivax malaria means that the latter species are more common causes of severe anemia and therefore more important targets for public health intervention. Nevertheless, on an individual basis, those with *P*. *malariae* parasitemia need to be investigated for anemia and treated aggressively if present. In this context, the relatively high rates of representation with *P*. *malariae* over the subsequent year (~4%), raises the possibility of partial response to ACT treatment coupled with prolonged subclinical carriage. There have been few studies of ACT drug efficacy in *P*. *malariae* and most have had only 28 days of follow-up [31]. Further efficacy studies with longer follow-up are warranted.

Nephrotic syndrome, a well-recognized complication of *P*. *malariae* infection, is mostly described in children living in endemic areas [32] with few cases reported since the mid-1970s [32–34]. Renal biopsies in two recent cases have shown chronic membranous glomerulopathy [34] and mesangioproliferative glomerulonephritis respectively [33]. The syndrome has been reported as being hard to treat and often unresponsive to corticosteroids, immunosuppressive agents and antimalarial drugs [35]. Given the lack of access to advanced diagnostic techniques in Timika, the diagnoses of nephrotic syndrome must be treated with caution. Moreover, we cannot infer that there was necessarily a causal relationship between the *P*. *malariae* infections and nephrotic syndrome. Nevertheless, we have, for the first time been able to estimate the risk of this condition at 1 in 200 presentations to hospital for children with *P*. *malariae* infection under the age of 5 years. None of the 4 patients with nephrotic syndrome and *P*. *malariae* infection were reported to have died during the follow-up period however 3 of the 16 patients who died were recorded as having chronic renal failure (not further specified) and one had acute renal failure. Unfortunately we did not have access to information on the presence or absence of albuminuria (a common finding in malariatherapy patients treated with *P*. *malariae* infections in the 1930s [16]).

A similar proportion of all patients presenting to hospital with *P*. *malariae* infection died as compared to patients with infection by the other *Plasmodium* species–a surprising finding given the supposedly low virulence of this species. The same was true of the subset of patients with *P*. *malariae* who were admitted to hospital. Based on the data at hand, we cannot make inferences on whether there was a causal link between the *P*. *malariae* infections and death. Fourteen of the deceased patients had a white cell count measurement during their final admission and in 9 cases, this was above the age-appropriate normal range. We were unable to find data on the typical impact of *P*. *malariae* infection on white cell counts. The high proportion of the deceased patients with leucocytosis could reflect the greater inflammatory response seen in severe malaria from all species [30, 36] and/or concomitant bacterial sepsis. Bacterial coinfection was certainly recorded in several cases. Severe anemia was only present in a quarter of the patients with *P*. *malariae* who died (40% if WHO criteria were used) and thus was not the sole attributable cause of death.

*Plasmodium malariae’*s tendency to cause prolonged asymptomatic and/or subpatent infections will confound elimination strategies that are based on active case detection or mass screening and treatment. Chronically infected patients with low-level parasitemia probably have substantial transmission potential and therefore mass drug administration campaigns could potentially have a significant impact on this species. Given the older age distribution of patients infected with *P*. *malariae*, it would be important to deliver the antimalarial drug to all age groups rather than just children.

Some important limitations of our study should be considered. Malaria diagnosis and species identification was based on microscopy alone (except for a small number of *P*. *falciparum* cases confirmed using rapid diagnostic tests), and parasitemia quantitation was not available. Previous studies have clearly demonstrated that microscopic diagnosis of *P*. *malariae* significantly underestimates the true prevalence of parasitemia when compared with PCR-based diagnosis and that correctly differentiating between *P*. *malariae* and *P*. *falciparum* based on morphology alone is fraught with error [37, 38]. A previous comparison between hospital and research laboratory microscopy results at RSMM showed 68.4% concordance in the diagnosis of *P*. *malariae* monoinfection with 5.3% of *P*. *malariae* cases actually deemed to be mixed *P*. *malariae/P*. *falciparum* infections and 26.3% of cases originally diagnosed as mixed *P*. *malariae* infections reclassified as *P*. *malariae* monoinfections [21]. *Plasmodium malariae* infection is often asymptomatic or minimally symptomatic, it is therefore likely that a smaller proportion of individuals infected with this species in the community presented to hospital for assessment and treatment when compared to infections by the other *Plasmodium* species. A potentially large ascertainment bias will therefore have occurred leading to an underestimation of the true prevalence and burden of *P*. *malariae* parasitemia in this region. Previous studies have shown that subpatent infection with *Plasmodium* species is associated with under-recognised morbidity (as well as ongoing risk of transmission), mostly in the form of chronic anemia [39–42].

The small fee charged to non-Papuans for care may have discouraged them from accessing the hospital services resulting in a degree of selection bias in our study. Consequently those of Papuan ethnicity were undoubtedly over-represented. Selection bias is also likely to have occurred in our analysis of hematological parameters as hemoglobin measurements were done according to the orders of the treating clinician rather than routinely in all patients. Patients without malaria were less likely to have had a full blood count. Since those who did not have a full blood count were presumably less likely to have been severely anaemic, our analyses will have underestimated the relative impact of malaria on hemoglobin concentrations in the study population. In exploring the anemia associated with *Plasmodium* infection we were unable to control for some potentially important confounders. We had no information on parasite quantitation/parasite biomass, the prevalence of hemoglobinopathies and red cell disorders such as G6PD deficiency or thalassemia and we were unable to control for factors such as malnutrition, iron deficiency and worm infestation, all of which are known to contribute significantly to anemia [43, 44]. It is highly probable that some of the anemia detected in those with *P*. *malariae* parasitemia was contributed to by these factors leading to an over-estimation of the species’ impact on hemoglobin concentrations. Any variations in the prevalence of these comorbid conditions in different regions will also limit the generalizability of our findings.

In conclusion, we have shown that although *P*. *malariae* is responsible for a small proportion of malaria infections in Papua, it is associated with appreciable morbidity and also mortality. This verifies the clinical relevance of infection with non-falciparum *Plasmodium* species and emphasizes the importance of attempting to eradicate all species infecting humans. The low parasite densities, high incidence of asymptomatic disease and the longevity of *P*. *malariae* infections are likely to present significant barriers to elimination of this species.

## Supporting Information

S1 Checklist STROBE checklist. (PDF)

S1 Data Database in tab delimited format. (TXT)
